# Epigenetic Predictor of Age

**DOI:** 10.1371/journal.pone.0014821

**Published:** 2011-06-22

**Authors:** Sven Bocklandt, Wen Lin, Mary E. Sehl, Francisco J. Sánchez, Janet S. Sinsheimer, Steve Horvath, Eric Vilain

**Affiliations:** 1 Department of Human Genetics, University of California Los Angeles, Los Angeles, California, United States of America; 2 Department of Biostatistics, University of California Los Angeles, Los Angeles, California, United States of America; 3 Department of Medicine, University of California Los Angeles, Los Angeles, California, United States of America; 4 Department of Biomathematics, University of California Los Angeles, Los Angeles, California, United States of America; 5 Center for Society and Genetics, University of California Los Angeles, Los Angeles, California, United States of America; University of Insubria, Italy

## Abstract

From the moment of conception, we begin to age. A decay of cellular structures, gene regulation, and DNA sequence ages cells and organisms. DNA methylation patterns change with increasing age and contribute to age related disease. Here we identify 88 sites in or near 80 genes for which the degree of cytosine methylation is significantly correlated with age in saliva of 34 male identical twin pairs between 21 and 55 years of age. Furthermore, we validated sites in the promoters of three genes and replicated our results in a general population sample of 31 males and 29 females between 18 and 70 years of age. The methylation of three sites—in the promoters of the EDARADD, TOM1L1, and NPTX2 genes—is linear with age over a range of five decades. Using just two cytosines from these loci, we built a regression model that explained 73% of the variance in age, and is able to predict the age of an individual with an average accuracy of 5.2 years. In forensic science, such a model could estimate the age of a person, based on a biological sample alone. Furthermore, a measurement of relevant sites in the genome could be a tool in routine medical screening to predict the risk of age-related diseases and to tailor interventions based on the epigenetic bio-age instead of the chronological age.

## Introduction

Throughout development, cells and tissues differentiate and change as the organism ages. This includes alterations to telomeres, accumulation of DNA mutations, decay of cellular and organ structures, and changes in gene expression [1]. Both differentiation of tissues, and ageing effects are at least partially caused by chemical modifications of the genome, such as DNA methylation.

Monozygotic (MZ) twins form an attractive model to study methylation changes with age. At the time of separation both embryos have nearly identical methylation patterns. While certain methylation changes are genetically controlled, environmental exposure and stochastic processes can lead to a change in methylation patterns. In this context, identical twins can be considered replicates of the same developmental and ageing experiment.

Several studies have investigated the epigenetic state of a small number of selected genes or CpG islands in subjects of varying age [2] or have measured the global changes in DNA methylation with increasing age [3]. Recently, unbiased genomewide studies have documented age effects on DNA methylation in cultured cells [4], mice [5], and humans [6], [7], [8]. Most of these reports' subjects were of a limited age range, and the continuity of the age related changes has been unclear. Therefore, estimating the age of a biological sample based on methylation balues has not been possible.

## Results

### Microarray analysis

In this study we quantified the methylation status of 27,578 CpG loci covering more than 14,000 genes at single-nucleotide resolution in saliva samples of 34 pairs of identical twins, between 21 and 55 years of age, using Illumina HumanMethylation27 microarrays. The twin pairs were recruited for a study on sexual orientation. No significant results for sexual orientation were found, which will be reported in detail elsewhere. Monozygosity was verified for all pairs by analysis of nine short tandem repeat probes. For each CpG site on the microarray, we calculated the beta value, which expresses the fraction of methylated cytosines in that location. A site that is completely methylated on both alleles in all cells has a beta value equal to 1; a completely unmethylated site equals 0. All subsequent analyses were performed on this beta value. For computational reasons, the data were filtered by requiring a mean methylation value between 0.05 and 0.95, and variance greater than 0. The resulting restricted dataset contained 16,155 probes, and all further analyses were performed on this filtered dataset. Batch effect were removed using the Combat algorithm [9], and one outlier sample was removed.

We first determined whether methylation differences measured using these arrays reflected actual differences between individuals by calculating the correlation coefficient between replicate arrays for 10 samples. The median correlation between replicate arrays was 0.995 (range 0.990–0.996), compared to 0.987 (range 0.957–0.994) between unrelated samples. This difference was highly significant (Wilcoxon test, *p* = 1.4×10^−7^). In unsupervised hierarchical clustering, the majority of twin pairs clustered together (Figure S1) and twin samples correlated with *r* = 0.992 (range 0.983–0.997), which is significantly different from the correlation between unrelated samples (Wilcoxon test, *p* = 1.93×10^−11^).

A previous study showed increasing global epigenetic differences with age in a sample of identical twins, suggesting increased epigenetic drift with age [3]. We were unable to replicate these genome-wide methylation changes when the intra-pair correlation coefficients, the intra-pair Euclidian distance, or the intra-pair Manhattan distance was correlated with age (*p*>0.1). We did, however, identify a subset of loci to be highly correlated with age.

**Figure 1:**
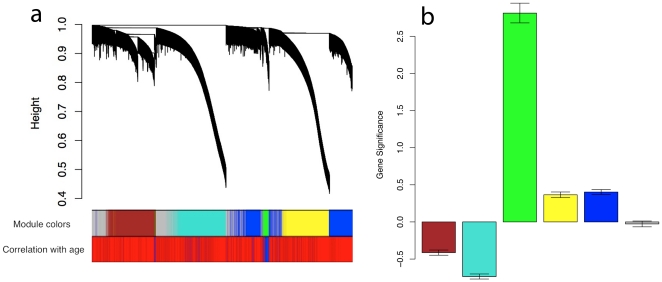
Detection of gene co-methylation modules in human saliva in twins. (a) Branches of the hierarchical cluster tree define five co-methylation modules which are assigned a color as can be seen from the first color band underneath the tree. Probes that could not be clustered into one of these modules were coded grey. Every probe represents a line in the hierarchical cluster tree. Distance between two probes is shown as height on the y-axis. The second color band encodes the age relationships of each gene. Genes with positive age correlations are colored in blue. (b) Barplots showing age relationships of modules. Specifically, the y-axis shows the mean Student T-statistic testing whether the methylation status of a probe is correlated with age. Note that the green module is enriched for probes that have a significant positive correlation with age. A t-statistic value of 2 or higher indicates a significant correlation (p<0.05).

A recurrent problem with data analysis on a whole genome scale is correcting for multiple comparisons. The stringency level of the chosen correction method strongly affects the odds of identifying significant findings. We previously described weighted correlation network analysis (WGCNA) as a data reduction scheme [10], [11]. Here we used WGCNA to identify modules of loci with highly similar methylation values. First, we averaged all methylation values for each twin pair, and treated each pair's data as an individual sample. Since both twins are genetically identical and of the same age, averaging the data reduces possible environmental effects on DNA methylation. After hierarchical clustering of the data set, branches of the cluster dendrogram defined five modules ranging in size from 199 to 842 loci, of which the methylation values were highly correlated across the samples (Figure 1A). We color coded the modules, calculated a weighted average, representative locus (eigenlocus) for each module (see Methods S1) and correlated this with age. The correlation between age and the representative of the green module was highly significant (*r* = 0.62, *p* = 7.2×10^−5^, Figure 1B), even after using the most stringent multiple comparison correction (Bonferroni), since only 5 comparisons—corresponding to 5 modules—were carried out. Module membership of all probes can be found in Dataset S1.

### Identification of 88 novel loci correlated with age

To identify novel loci for which the methylation values correlate positively or negatively with age, we calculated *q*-values to correct for multiple comparisons [12]. We selected probes with *q*-values smaller than 0.05, corresponding to absolute correlation values greater than 0.57. A total of 88 probes correlated with age (Table S1), corresponding to 80 genes spread over several of the modules. Of these, 19 probes were negatively correlated, and 69 were positively correlated with age, of which 57 belonged to the green module. A recently published study used a very similar study design and identical microarrays to identify 131 CpG sites correlated with age in blood samples of identical twins ranging from 49 to 75 years of age [8]. Of these 131 sites, 10 were found to be positively correlated with age in our study as well (Table S2).

Of the 88 probes that were significantly correlated with age in our study, only one was near a gene encoding a microRNA (HSA-MIR-10A, in the HOXB4 gene), which was not different from the density on the array. 73 of 88 (83%) significant probes were within CpG-islands, thus this probe set was enriched in CpG islands relative to the typical array probe (73% in CpG islands, *p* = 0.031, Fisher's exact test for count data). CpG sites that were significantly correlated with age were a median 238 base-pairs upstream of the transcription start site.

Ingenuity analysis showed the 80 age-related genes were highly enriched for genes involved in cardiovascular disease (*p* = 1.59×10^−6^), neurological disease (*p* = 1.47×10^−4^), and genetic disease (*p* = 1.59×10^−6^)—a category consisting almost entirely of the cardiac and neurological genes as well. The most enriched cellular function was molecular transport (*p* = 2.4×10^−3^). The full gene ontology analysis can be found in Table S3.

### Validation of correlated probes in additional samples

**Figure 2:**
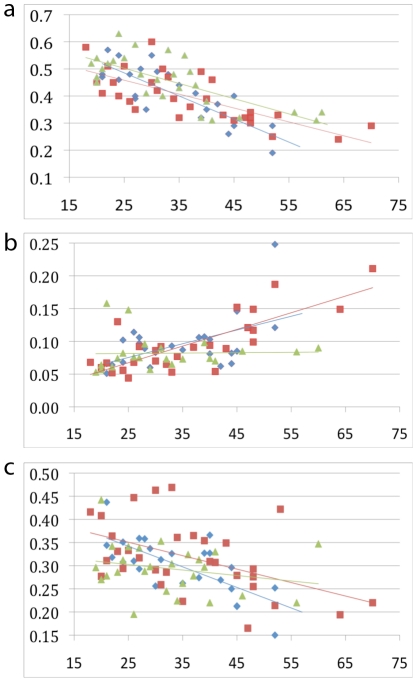
Percentage methylation versus age for three markers validated in three sample sets. Original twin samples are blue, male control samples are red, female control samples green. Linear trendlines are shown in the colors of the individual sample sets a) Edaradd *r* = −0.81 (twins), *r* = −0.73 (male controls), *r* = −0.75 (female controls) b) NPTX2 *r* = 0.52 (twins), *r* = 0.79 (male controls), *r* = 0.03 (female controls) c) Tom1L1 *r* = −0.70 (twins), *r* = −0.49 (male controls), *r* = −0.24 (female controls).

Three probes for which the methylation status was highly correlated with age, and which had the widest distribution of values, were chosen for further validation. Saliva samples from 22 twins from the array study, 31 unrelated male, and 29 unrelated female samples (age range  =  18–70 years-old) were bisulfite converted and PCR amplified. The fraction of methylated cytosines at the exact CpG sites assayed on the Illumina arrays were quantified by MassArray (Sequenom) for the Edaradd gene and by pyrosequencing for NPTX2 and Tom1L1. For NPTX2, the pyrosequencing method provided methylation data for five additional CpG sites in the promoter. The results of the validation experiments correlated very strongly with the array data for all three genes (Edaradd *r* = 0.96, NPTX2 *r* = 0.92, Tom1L1 *r* = 0.90, *n* = 23), providing a technical replication of the array data in the twin sample. The correlation between the degree of methylation and age of all three genes was preserved in the subset of twins and was also found in the independent male sample, providing a biological replication. In females, Edaradd and Tom1L1 were significantly correlated with age, but NPTX2 was not. The correlation results are shown in Figure 2. A multivariate linear regression model using Edaradd, Edaradd squared and NPTX2 showed that these two markers explain 76% (or *R* ^2^ = 0.76) of the variance in age of males and 70% in females. When considering males and females together the model explained 73% of the variance in age.

### A leave-one-out analysis forms an accurate epigenetic predictor of age

**Figure 3:**
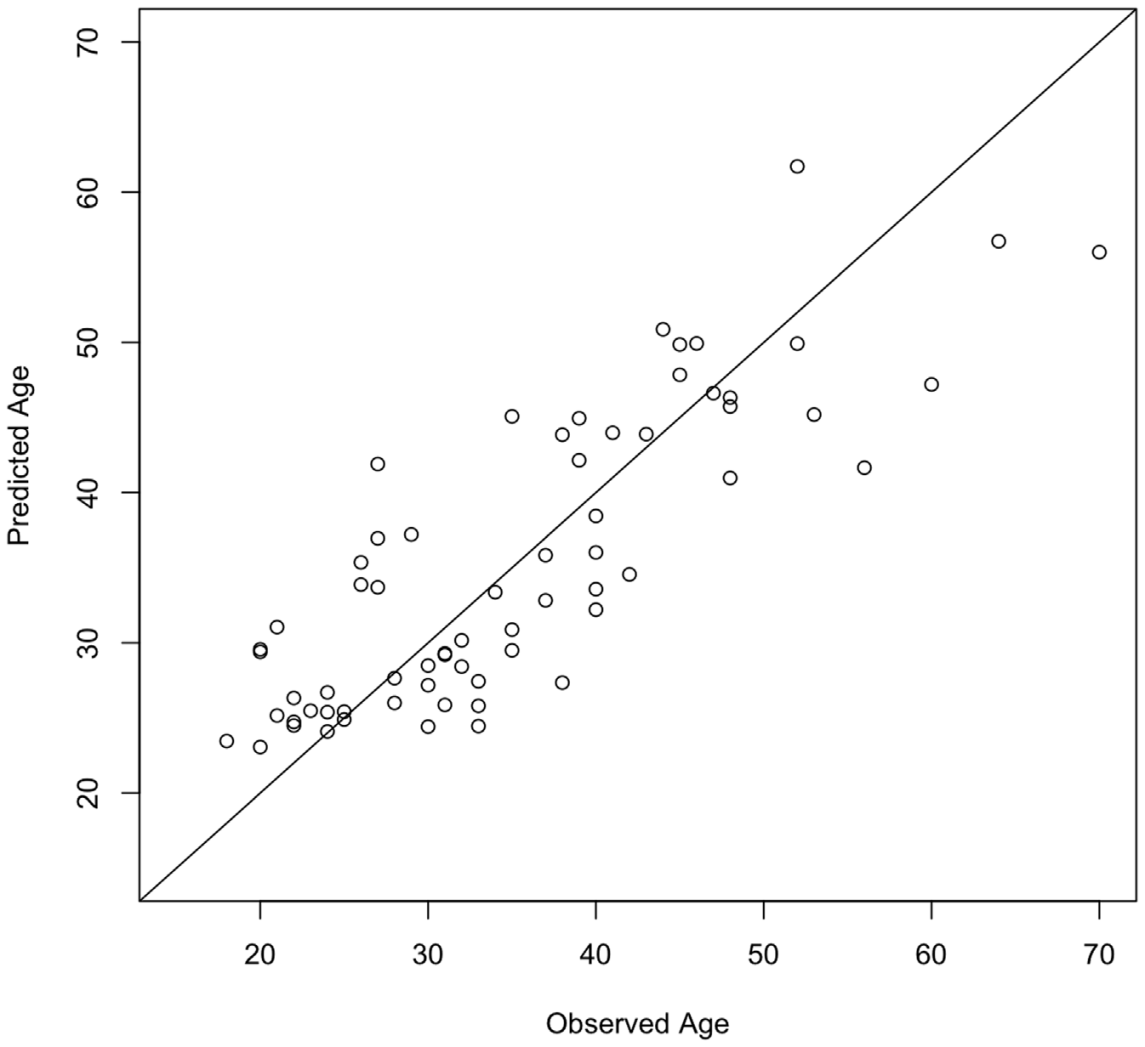
Predicted versus observed age of all subjects using a leave-one-out model. A multivariate regression model was fit on all but one sample and its predicted age (y-axis) was related to the truly observed age of the left out sample (x-axis). The predicted values are highly correlated with the observed ages (r = 0.83, p = 2.2×10^−16^, n = 66), and the average absolute difference between the predicted and the observed age is 5.2 years.

To provide an unbiased estimate of predictive accuracy for age, we used a leave-one-out analysis where the multivariate regression model was fit on all but one subject and its prediction was related to the truly observed age of the left-out subject. The predicted values are highly correlated with the observed age in males (*r* = 0.83, *p* = 3.3×10^−13^, *n* = 47, Figure S2), females (*r* = 0.75, *p* = 2.4×10^−4^, *n* = 19, Figure S3), and in the combined sample (*r* = 0.83, *p* = 2.2×10^−16^, *n* = 66, Figure 3). For the male only or female only models, the average absolute differences between the predicted and the observed age (the error) are 5.3 years and 6.2 years, and for the combined sample this is 5.2 years. Even when only the male and female replication samples were used, discarding all twin data, the accuracy of the model remained at 5.3 years, and the predicted values correlated highly with the observed age (*r* = 0.85, *p* =  1.701×10^−13^, *n* = 45, Figure S4).

To test whether additional data points on the microarray could improve the accuracy of the model, we performed lasso penalized regression to screen for the top predictors of age [13], [14]. The top five predictors were tested, and only three were found to contribute significantly to the regression model: Edaradd, NPTX2, and ELN. The first two predictors were already part of the model. Using the microarray methylation data for these two genes, the average error is 4.7 years (*r* = 0.77, *p* = 1.029×10−07, *n* = 34). Adding the ELN methylation data improved the accuracy of our model, reducing the average error to 3.5 years (*r* = 0.87, *p* = 2.2×10−11, *n* = 34, Figure S5). Results were nearly identical when all twin samples were treated as unrelated individuals, and when averaged values for each pair were used. The distribution of methylation values for ELN was considered too narrow for further validation using pyrosequencing or MassArray analysis.

## Discussion

In this high density, genomewide screen of CpG methylation of twins, we identified 88 CpG sites near 80 genes for which the percent methylation in saliva is significantly correlated with age. These are highly enriched for genes known to influence age-related diseases—mainly cardiovascular and neurological disease. Ten of these 88 CpG sites were shown earlier to be correlated with age in whole blood and in isolated CD4^+^ and CD14^+^ cells as well [8]. We validated three genes in a sample of unrelated males and females, which confirmed our findings in these replicate samples. Remarkably, the methylation values for the validated genes are linear with age over a span of five decades and in three separate sample sets. Based on this observation, we were able to build a model that can predict the age of a subject based on the methylation status of just two cytosines in the genome, explaining 73% of the variance in age.

Of the validated genes, Neuronal Pentraxin II (NPTX2) methylation has been shown to be upregulated in pancreatic cancer [15], and its expression is increased in Parkinson's disease [16]. Its methylation status was recently shown to be correlated with age in blood as well [8]. Mutations in the Edar associated death domain (Edaradd) can cause loss of hair, sweat glands, and teeth [17], and it can reduce the speed of wound healing [18]. Further research should focus on their role in ageing, and age-related diseases.

The lack of epigenetic drift within each monozygotic pair contrasts with a previous study [3]. The main difference between the two studies is that we focused on CpG sites close to functional gene transcription start sites whereas Fraga and colleagues investigated random sites, most of which were located in non-functional repeated sequences (e.g., Alu repeats). This suggests that while drift may occur randomly with age in non-coding, repeat-rich DNA regions, the critical regulatory portions of the genome remain under strict epigenetic control throughout life.

Our regression model (Figure 3) could be applied in a variety of contexts. For instance, our ability to predict an individual's age to an average accuracy of 5.2 years could be used by forensic scientists to estimate a person's age based on a biological sample alone, once the model has been tested in various biological tissues. The model is also relevant to healthcare applications. Previously, significant DNA methylation differences were shown to be associated with specific age-related disorders, for example in comparisons between the brains of people diagnosed with late-onset Alzheimer's disease and brains from controls [19]. The identification of specific epigenetic patterns highly correlated with age has the potential to influence our understanding of ageing in health and disease. Specifically, it could lead to clinical interventions that are tailored to patients based on their “bio-age”—a result of the interaction of genes, environment, and time—rather than their chronological age. Future investigations should focus on phenotype and disease history of those subjects whose predicted age vary widely from their actual age. Furthermore, these findings could pave the way for interventions based on specific epigenetic marks associated with disease, as is already the case in cancer treatment [20].

## Materials and Methods

### Ethics statement

The study was approved by the UCLA Institutional Review Board, and all subjects signed informed consent.

Monozygotic twin pairs, differing for sexual orientation, were recruited through the study website, online advertisement and press coverage. Male and female control subjects were recruited using fliers. There were no significant differences in racial composition between the sample sets or age groups. Saliva was collected using Oragene DNA collection kits (Genotek). The majority (up to 74%) of the DNA in saliva collected with this method typically comes from white blood cells, with the remainder being buccal epithelial cells [21]. Genomic DNA was prepared according to the manufacturer's protocol. Zygosity was determined using 9 microsatellite markers. Microarray hybridization was performed by the Southern California Genotyping Consortium at UCLA. 500 ng of genomic DNA was bisulfite converted using the EZ-methylation kit (Zymo Research), and processed according to the Illumina Infinium whole genome genotyping protocol. Labeled samples were hybridized to Illumina HumanMethylation27 arrays, scanned (iScan reader, Illumina), and beta (methylation) values extracted using GenomeStudio software. All array data is MIAME compliant, and the raw data has been deposited in NCBI's GEO, a MIAME compliant database as detailed on the MGED Society website (http://www.mged.org/Workgroups/MIAME/miame.html) under accession number GSE28746.

Analysis: A signed weighted correlation network was constructed as described [11], [22]. Module definition was based on the gene methylation status in saliva and ignored age. As module representative, we used the module eigenlocus (ME) which is defined as the first principal component of the module methylation profiles and can be considered a weighted average. To incorporate age into the network analysis, the Student t-test statistic for correlating age with methylation status was used. Lasso penalized regression was performed using the ‘penalized’ package of R[14]. All statistical analyses and data processing were performed using the statistical package R version 2.11.1 [23]. PCR reactions for amplification, massarray and pyrosequencing analysis were performed using Sahara and Bio-X-ACT Long enzymes (Bioline). PCR primers and conditions are listed in Methods S1.

## Supporting Information

Data Set S1 Full statistics and module membership of all array probes.
(3.01 MB XLS)

Table S1 88 loci significantly correlated with age TargetID represents the exact Illumina probe on the array, Chr: chromosome number, Gene_ID: NCBI Gene database locator, Symbol: gene name, r: correlation coefficient, p-value: significance of correlation, q-value: significance corrected for multiple comparisons.
(0.04 MB XLS)

Table S2 Array probes found to be positively correlated with age in blood (published data) and in saliva (present study).
(0.04 MB XLS)

Table S3 Disease and molecular function categories significantly enriched in ingenuity analysis.
(0.03 MB XLS)

Figure S1 Unsupervised hierarchical clustering of all samples. The y-axis shows distance between samples. Each twin pair is color coded. Row "Pair" shows that the majority of twin pairs cluster together. Samples were divided in the oldest and youngest half and coded dark and light blue. Row "Age" shows that samples of similar age group did not cluster together. The different arrays were each color coded as well, and row "Array" shows that samples hybridized together do not cluster together, suggesting that variations in hybridization do contribute to the data analysis.
(6.75 MB TIF)

Figure S2 Predicted versus observed age of all male subjects using a leave-one-out model. A multivariate regression model was fit on all but one sample and its predicted age (y-axis) was related to the truly observed age of the left out sample (x-axis). The predicted values are highly correlated with the observed outcomes (r = 0.83, p = 3.3×10^−13^, n = 47), and the average absolute difference between the predicted and the observed age is 5.3 years.
(4.31 MB TIF)

Figure S3 Predicted versus observed age of all female subjects using a leave-one-out model. A multivariate regression model was fit on all but one sample and its predicted age (y-axis) was related to the truly observed age of the left out sample (x-axis). The predicted values are highly correlated with the observed outcomes (r = 0.75, p = 2.4×10^−4^, n = 19), and the average absolute difference between the predicted and the observed age is 6.2 years.
(4.30 MB TIF)

Figure S4 Predicted versus observed age of all non-twin subjects using a leave-one-out model. A multivariate regression model was fit on all but one sample and its predicted age (y-axis) was related to the truly observed age of the left out sample (x-axis). The predicted values are highly correlated with the observed outcomes (r = 0.85, p =  1.701×10^−13^, n = 45) and the average absolute difference between the predicted and the observed age is 5.3 years.
(4.30 MB TIF)

Figure S5 Predicted versus observed age of all twin subjects using a leave-one-out model. A multivariate regression model was fit on data of previously used markers plus the methylation value at the ELN gene, on microarray data, for all but one sample and its predicted age (y-axis) was related to the truly observed age of the left out sample (x-axis). The predicted values are highly correlated with the observed outcomes (r = 0.87, p = 2.2×10^−11^, n = 34), and the average absolute difference between the predicted and the observed age is 3.5 years.
(4.30 MB TIF)

Methods S1 PCR protocol and primers.
(0.03 MB DOC)
