# Supplementary figures and images for: Epigenetic Predictor of Age
Source: PLoS One. 2011 Jun 22;6(6):e14821. doi: 10.1371/journal.pone.0014821 (PMC3120753; doi:10.1371/journal.pone.0014821)

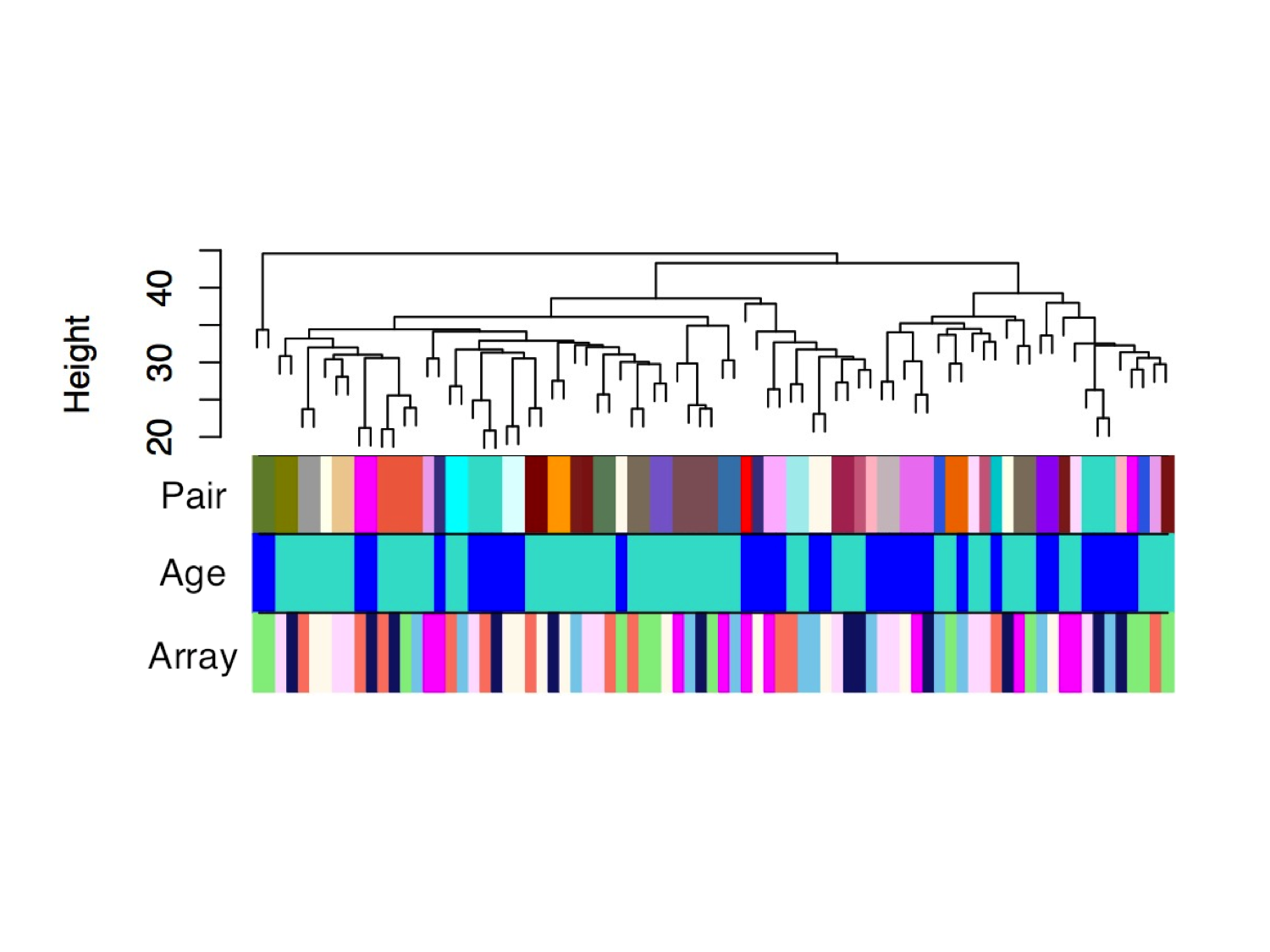

Supplement: Figure S1 — Unsupervised hierarchical clustering of all samples. The y-axis shows distance between samples. Each twin pair is color coded. Row "Pair" shows that the majority of twin pairs cluster together. Samples were divided in the oldest and youngest half and coded dark and light blue. Row "Age" shows that samples of similar age group did not cluster together. The different arrays were each color coded as well, and row "Array" shows that samples hybridized together do not cluster together, suggesting that variations in hybridization do contribute to the data analysis. (6.75 MB TIF) [file pone.0014821.s005.tif]

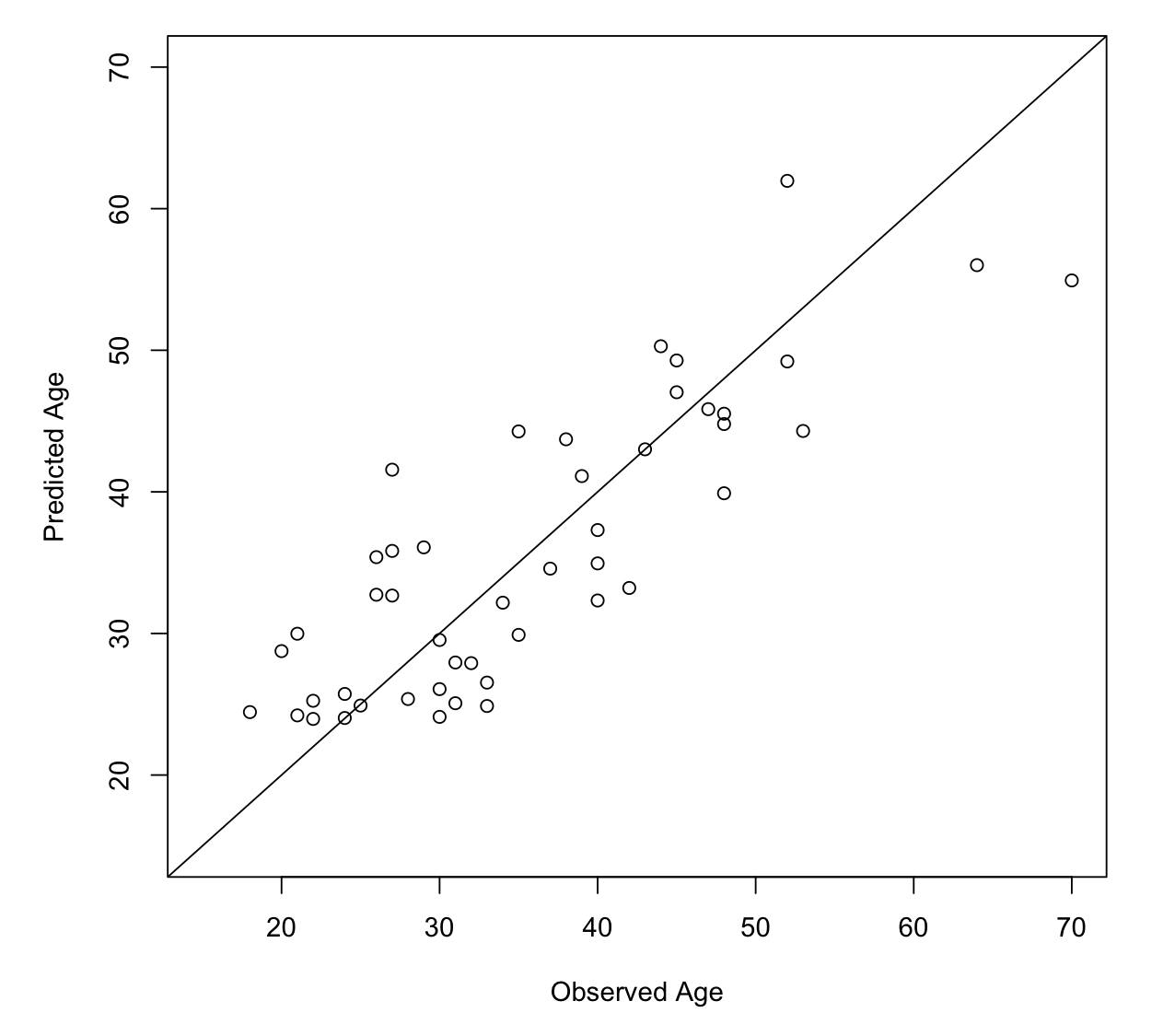

Supplement: Figure S2 — Predicted versus observed age of all male subjects using a leave-one-out model. A multivariate regression model was fit on all but one sample and its predicted age (y-axis) was related to the truly observed age of the left out sample (x-axis). The predicted values are highly correlated with the observed outcomes (r = 0.83, p = 3.3×10^−13^, n = 47), and the average absolute difference between the predicted and the observed age is 5.3 years. (4.31 MB TIF) [file pone.0014821.s006.tif]

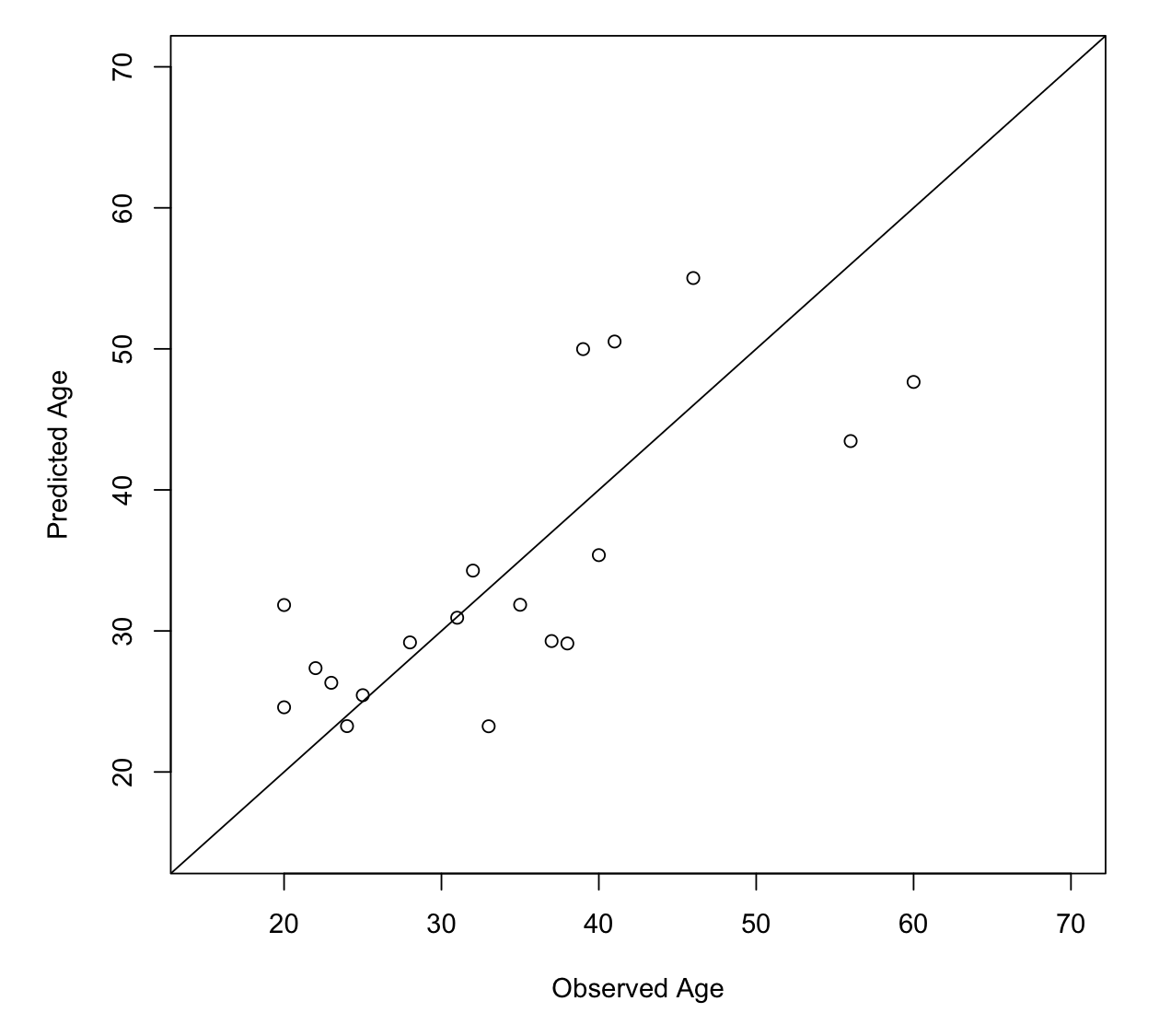

Supplement: Figure S3 — Predicted versus observed age of all female subjects using a leave-one-out model. A multivariate regression model was fit on all but one sample and its predicted age (y-axis) was related to the truly observed age of the left out sample (x-axis). The predicted values are highly correlated with the observed outcomes (r = 0.75, p = 2.4×10^−4^, n = 19), and the average absolute difference between the predicted and the observed age is 6.2 years. (4.30 MB TIF) [file pone.0014821.s007.tif]

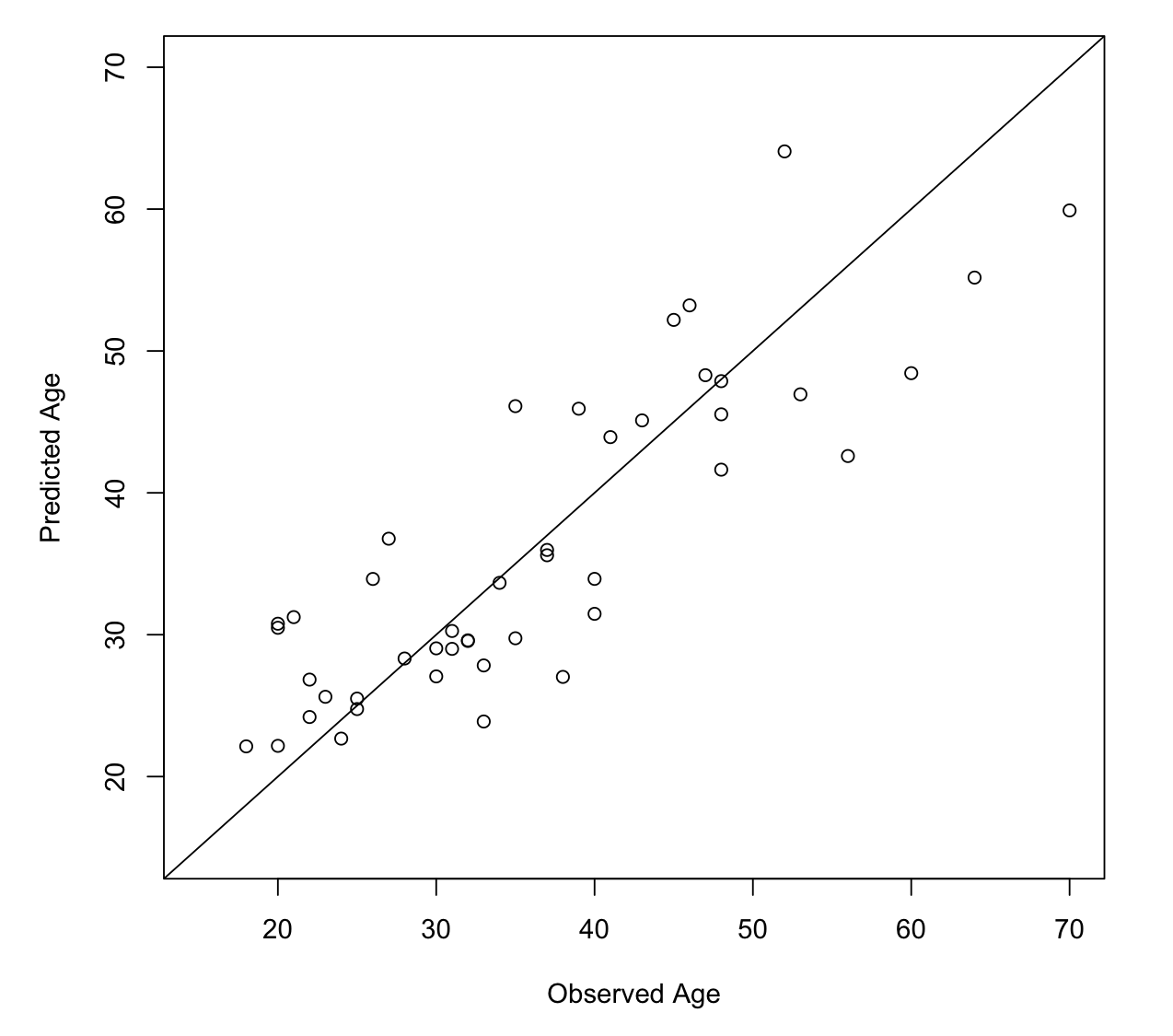

Supplement: Figure S4 — Predicted versus observed age of all non-twin subjects using a leave-one-out model. A multivariate regression model was fit on all but one sample and its predicted age (y-axis) was related to the truly observed age of the left out sample (x-axis). The predicted values are highly correlated with the observed outcomes (r = 0.85, p = 1.701×10^−13^, n = 45) and the average absolute difference between the predicted and the observed age is 5.3 years. (4.30 MB TIF) [file pone.0014821.s008.tif]

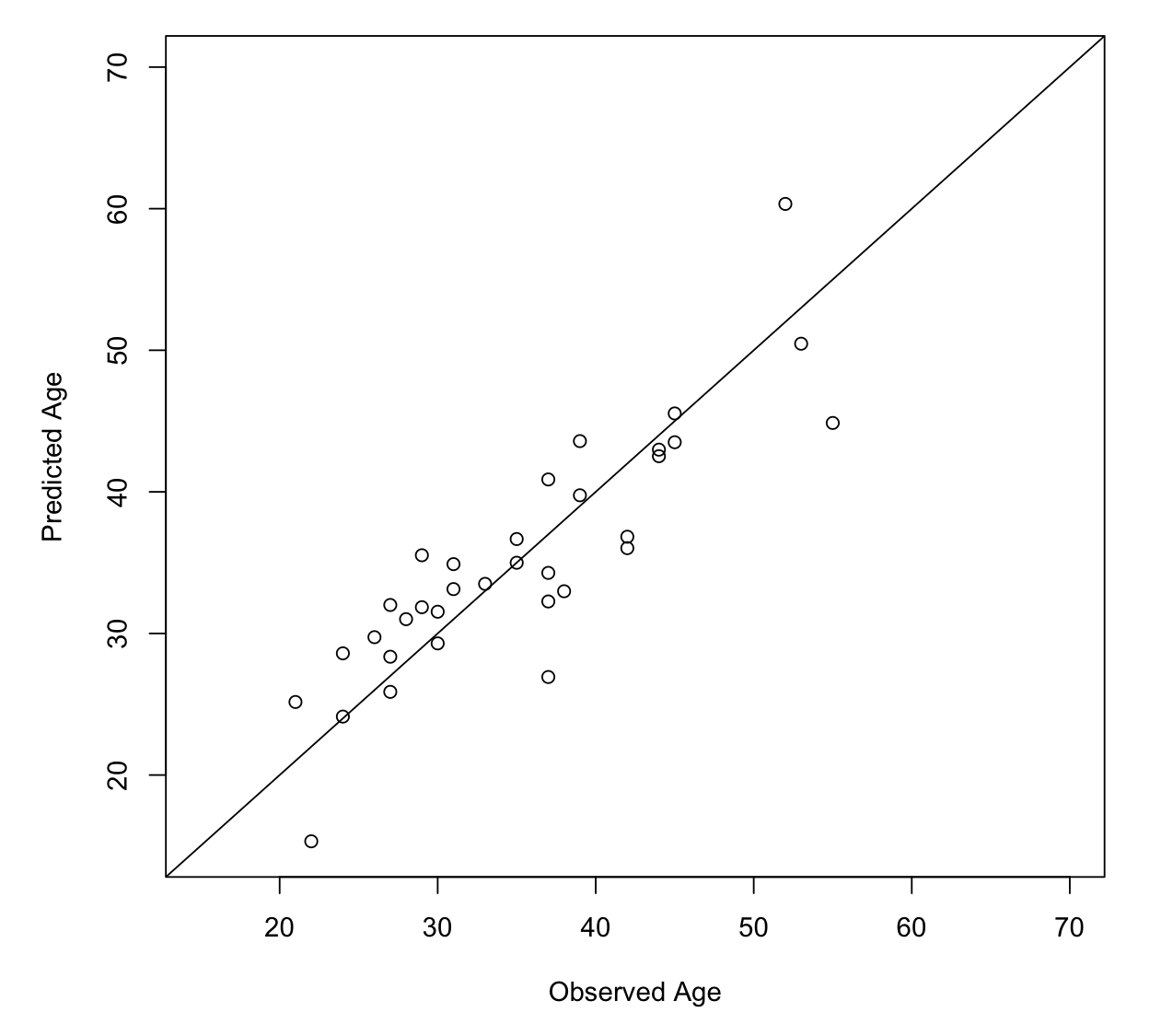

Supplement: Figure S5 — Predicted versus observed age of all twin subjects using a leave-one-out model. A multivariate regression model was fit on data of previously used markers plus the methylation value at the ELN gene, on microarray data, for all but one sample and its predicted age (y-axis) was related to the truly observed age of the left out sample (x-axis). The predicted values are highly correlated with the observed outcomes (r = 0.87, p = 2.2×10^−11^, n = 34), and the average absolute difference between the predicted and the observed age is 3.5 years. (4.30 MB TIF) [file pone.0014821.s009.tif]
