# Supplementary material for: Epigenetic Predictor of Age
Source: PLoS One. 2011 Jun 22;6(6):e14821. doi: 10.1371/journal.pone.0014821 (PMC3120753; doi:10.1371/journal.pone.0014821)
Supplement: Methods S1 — PCR protocol and primers. (0.03 MB DOC) [file pone.0014821.s010.doc]

**SUPPORTING METHODS**

**PCR protocol and primers**

500 ng of genomic DNA was bisulfite converted using the EZ-methylation kit (Zymo Research), and 10 ng was used for each reaction. Edaradd was amplified using Bio-X-ACT Long (Bioline) with 20 seconds annealing at 57C, and 30 seconds extension at 68C, for 40 cycles. Tom1L1 and NPTX2 were amplified using Sahara Mix (Bioline) with 20 seconds annealing at 58C (Tom1L1) or at 55C (NPTX2), and 25 seconds extension at 72, for 40 cycles. Primer sequences for the amplicons are listed below. The appropriate sequence tags were added to the Edaradd PCR primers according to the instructions provided by Sequenom.

Edaradd methylation was assayed using MassArray (Sequenom) by the Genomics Core of the Albert Einstein College of Medicine, and the percent methylation at each CpG site extracted with EpiTyper software (Sequenom).

Tom1L1 and NPTX2 methylation was assayed using Pyrosequencing by the UCLA Genotyping and Sequencing Core.

Edaradd:

F: GGTAGATTAAGAGGAAGTTTATTTTTTTAT

R: AATACCTCTCCCCATCTATTTAATC

Tom1L1:

F: TTAATTTATTGTAGAATTTT

R: AAACCTCCTCTTCTAATCTATAAAAC

Sequencing primer: ATAAAATATTTAAACCTCCA

NPTX2:

F: TAGTTTAAGAAAGGG

R: AACTATCCTAAACCCCAAC

Sequencing primer: ACAAAAAACTTCTACCC
